# A Data-Driven Personalized Lighting Recommender System

**DOI:** 10.3389/fdata.2021.706117

**Published:** 2021-10-14

**Authors:** Atousa Zarindast, Jonathan Wood

**Affiliations:** Department of Civil and Environment Engineering, Iowa State University, Ames, IA, United States

**Keywords:** smart recommendation system, lighting, color prediction, routine recommender, personalization

## Abstract

Recommender systems attempt to identify and recommend the most preferable item (product-service) to individual users. These systems predict user interest in items based on related items, users, and the interactions between items and users. We aim to build an auto-routine and color scheme recommender system for home-based smart lighting that leverages a wealth of historical data and machine learning methods. We utilize an unsupervised method to recommend a routine for smart lighting. Moreover, by analyzing users’ daily logs, geographical location, temporal and usage information, we understand user preferences and predict their preferred light colors. To do so, users are clustered based on their geographical information and usage distribution. We then build and train a predictive model within each cluster and aggregate the results. Results indicate that models based on similar users increases the prediction accuracy, with and without prior knowledge about user preferences.

## 1 Introduction

The technological revolution has facilitated the rise of smart ecosystems where every aspect of everyday life such as transportation, agriculture, logistics, and healthcare are automated and can be controlled and managed in the context of smart cities Ahad et al. (2020). Smart cities, assisted by modern digital technologies, are a potential solution to enhanced quality and performance of urban service Sikder et al. (2018).

The introduction of Internet-of-Things (IoT) in smart cities brings opportunities to interconnect different applications using information and communication technology. One of the many important implementations of IoT systems is lighting systems. Lighting systems account for approximately one-third of electricity usage in commercial buildings Ryckaert et al. (2010). One of many potentials of smart lighting systems is energy saving capabilities. Systems with integrated energy saving light control systems can have 17–90% energy savings over traditional lighting systems Von Neida et al. (2001). Beyond energy, smart lighting can be used to increase light quality and have a positive impact on productivity Karlicek (2012), and circadian rhythm Oh et al. (2014).

Light emitting diodes (LEDs) can be used for illumination and decoration purposes, and dimming capability provides greater control over the light product. Furthermore, LEDs have low power consumption and long lifespans, making them ideal for multi-channel lighting systems. The future of smart lighting research is multi-disciplinary with potential improvements in energy efficient buildings, building management systems, smart cities, human health, and psychology. The application of smart lighting and control systems has been widely analyzed in industrial settings; however, the application and usage in household residencies are relatively new. Smart lighting has potential for improving house lighting; however, there are barriers to the adoption rates among potential users due to the initial investment cost. There is room for improving the functionality of smart lighting systems to increase the attractiveness to current users and potential buyers. It has been shown that ease of use and control-ability are important contributing factors among people who purchased smart lighting products Zarindast et al. (2021a) and among potential smart lighting customers Shin et al. (2018).

Recent advancements in artificial intelligence, computational power, storage capacity, and edge computing has made it possible to leverage historical and real-time information to learn patterns and provide solutions including recommender systems. Recommender systems can be used to improve user satisfaction, increase usability, and improve the control-ability of smart products. These systems account for user preferences based on historical and real-time information. In this paper, we introduce a light routine and color recommendation system for household residents. This recommendation system is intended as an add-in feature to current and future smart lighting systems. For this purpose, we develop a machine learning model that leverages historical log data to learn and propose personalized recommendations. We then built a context-aware predictive model based on geographical, temporal, and usage data of lights in households using machine learning. The end product of this system is a light color recommendation, defined as “scene”. These models are based on worldwide user logs of a smart lighting system. The accuracy and robustness of the system was tested using data from users across multiple countries. Our solution is robust by sampling a diverse and wealthy amount of data in terms of time period and number of households as its metrics show low bias and high confidence levels.

## 2 Literature Review

The literature review is separated into two categories of research, namely light control systems and recommendation systems. This paper proposes a data-driven personalized light recommendation system in a smart lighting device adjusted to residential requirements. There are numerous studies conducted on light control systems for office requirements that may not suit household needs due to the differences in intended uses and needs of the lighting systems.

### 2.1 Light Control Systems

Lighting can contribute to improve mood and productivity while maintaining comfort and increasing satisfaction Juslén et al. (2007); Van Den Wymelenberg and Inanici (2014); Boyce et al. (2000). A significant amount of people’s time is spent indoors; hence, one of the main objectives of building control systems should be to provide indoor comfort. However, building control systems usually neglect to directly consider occupant satisfaction in lighting design criteria Park and Nagy (2018). One aspect of comfort is defined as having control over the indoor environment while interacting with the system Nagy et al. (2016).

Occupant based control systems are a feasible solution for automated control systems in work places. There are several occupancy based light control system studies that focus specifically on workplaces. For example, Caicedo et al. (2011) suggested a light control system that considers occupancy and locations of the occupants to provide optimum brightness levels. Peruffo et al. (2015) proposed a wireless network lighting system with multiple sensors that determines daylight levels and occupancy along with a central controller. The control system’s output is a dimming level for lights based on occupancy and daylight. Gunay et al. (2017) proposed a lighting and blind control algorithm for office environment. Moreover, they analyzed occupant behavior with different scenarios and simulations. These models automatically control lighting systems but neglect human perception, mood, comfort and other occupant preferences. Given the importance of user satisfaction and comfort, research on both comfort and control systems has emerged Heydarian et al. (2015); de Korte et al. (2015).

Recent studies have evaluated lighting control systems based on occupancy and have considered occupant comfort in their modeling for office layouts. Nagy et al. (2015) introduced an occupancy based lighting control system based on statistical analysis of sensor data. They identified minimum and maximum brightness threshold levels by interacting with the user based on statistical data analysis. Cheng et al. (2016) proposed a closed loop satisfaction based Q-learning control system that receives users’ perception as feedback signals. In the proposed Q-learning based system, users’ explicit feedback and interaction with the system is required. User feedback is a useful source for modeling but is not always practical to incorporate into the system. Users may not be willing to take the time to give feedback for the system, particularly in home based environment. Therefore, implicit understanding of user preferences while interacting with the system is a more practical and feasible solution in residential settings.

Zou et al. (2018) proposed a smart lighting control system that adjusts the brightness level of lamps based on real time occupancy data to minimize energy consumption. They included personalization by an app control feature that enables the occupant to adjust the brightness level of a nearby lamp. They conducted an experiment over a 24 weeks’ time period and evaluated the performance based on occupancy detection accuracy and energy savings. In their proposed model, the desired brightness level of each occupant is a given parameter to an optimization problem and does not reflect the dynamics in mood, individual preferences, and changing requirements for different activities. Park et al. (2019) introduced a reinforcement learning based control system for office location that is based on occupancy. They collected data from five offices over an 8 week period. All of the above mentioned studies propose a control system for a business framework that would not be suitable for household requirements yet are helpful to understand.

### 2.2 Recommendation Systems

Recommendation systems can be defined as systems that attempt to identify and recommend the most preferable item (product-service) to individual users. These systems predict user interest in items based on related items, users, and the interactions between items and users (Lu et al., 2015). Commonly used recommendation techniques include collaborative filtering Burke (2000) which suffers from sparseness, scalability and cold start problems Adomavicius and Tuzhilin (2005); and content based Pazzani and Billsus (2007) techniques which have overspecialized recommendation applications. Content based filtering primarily extracts the content as a basis for item prediction and attempts to build a user profile using preference indicators van Capelleveen et al. (2019). There are numerous examples of recommender system applications including E-library Porcel and Herrera-Viedma (2010), E-commerce Lawrence et al. (2001), movie, video Lee et al. (2010) and TV program Kwon and Hong (2011) recommenders. To date, however, no studies have provided recommendation systems or investigated machine learning to recommend residential light usage routines and scenes.

One of the challenges for recommendation systems is the cold-start problem due to a lack of prior knowledge specific to an individual new user. To deal with the cold-start problem, researchers have recently considered social media as a source of understanding customer characteristics and traits. Cho et al. (2020) included what is called lifelog information in smart lighting control system. Lifelogs include personal information related to a user’s activities, biometrics and environmental information. They included the user’s message, app location, activity, and weather data in their analysis and introduced a lighting control system. Their system was developed for a single household using an infrastructure consisting of motion sensor, pressure sensor on seats, an IoT camera in the kitchen for taking pictures of the food consumed by the user. Moreover, to understand the user’s emotions, they utilized text message analysis. Although lifelog information can provide a customized and personalized setting for each user, it has several limitations: 1) it has to be compiled for each user separately, 2) it may have scalability issues due to large-scale implementation of such infrastructure not always being practical, 3) there may be privacy issues related to gathering this kind of information, as users may not feel comfortable with being monitored at this level. Therefore, a more generic personalized system is needed to propose personalized recommendations that respect user privacy and do not require complex infrastructure for implementation.

This paper, investigates the application of historical usage logs to build a data-driven recommender system for household lighting systems. The aim is to increase the utility of the lighting system and introduce advanced smart lighting features that can learn from household users’ past behaviors and incorporate user preferences in the recommender system. User perceptions are investigated implicitly by leveraging past historical data. In this manner, our system applies an implicit understanding of user behaviors and preferences to take charge of their environment via increased control over the lighting system. Recommended preferences and increased control over the lighting system has the potential to improve the user experience Zarindast et al. (2021a).

## 3 Methodology

### 3.1 Data Abstraction

The mathematical abstraction for describing the data is presented in this section. The data set is based on actions (e.g., turn on/off, color change, rule setting, etc.,) and each observation defines an order that targets a smart light bulb. Light orders can come from various sources such as app, buttons, and switches. All the orders go through a device called a hub which is responsible for the light-user interactions and for saving the communications between light bulb and the hub as log entries. Important features of each order considered in this analysis include timestamp associated with each order, color dimension features (saturation, brightness, color coordinates (x,y), color temperature, color mode, light id, group id that defines room type, and hub id which is the unique identification of a household, city and country. Overall this results in a 5th order tensor:

$$X=\left\{x^{t_{1},d,r,b,s},x^{t_{2},d,r,b,s},\ldots ,x^{t_{N},d,r,b,s}\right\}$$ (1)

Where *x* is a Boolean variable (on/off), *t*_*i*_ denotes the *ith* time instance in which *T* = { *t*_*i*_∣*i* ∈ *N* }, *s* ∈{*S*} denotes scenes, *d* ∈{*D*} denotes days, *r* ∈{*room*1, *room*2} denotes room type, *b* ∈{*B*} denotes households.

### 3.2 Routine Recommender

**FIGURE 1:**
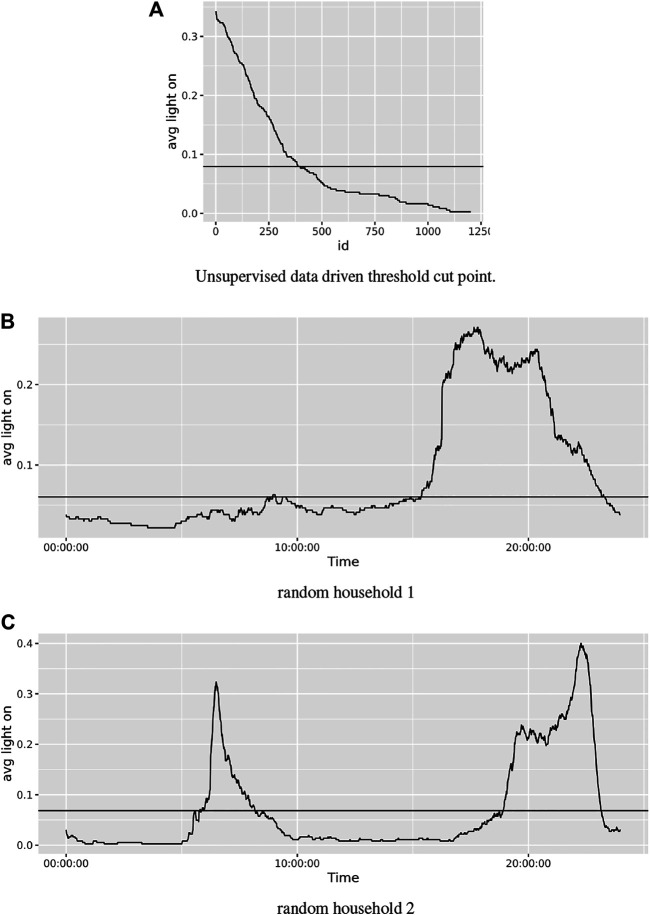
Usage distribution and cutoff point.

Our framework is separated into two major sub-systems, namely 1) routine and 2) scene recommenders. The routine recommender is based on the frequency of light usage in each room type. We utilize an unsupervised learning method to select highly used periods of time. The frequency of use in each timestamp is defined using Eq. 2 and is shown in Figure 1. Using the elbow cutoff method, as described in previous studies Zarindast et al. (2021b) (submitted); Zarindast (2019) , shown in Figure 1A we define the margin in which the lights are highly used throughout the time period. The horizontal line in Figure 1 represents the margin identified by the elbow cutoff point, and the timestamps that have a frequency above the threshold point are the desired period of time based on usage. For instance, the recommended routine for this particular user in this room type occurs in the 7–10 PM time slot.

$$\;avg\;light\;on\;frequency=\frac{\overset{D}{\underset{d=1}{\sum }}on}{D},\forall t_{i}\in N$$ (2)

### 3.3 Scene Recommender

**FIGURE 2:**
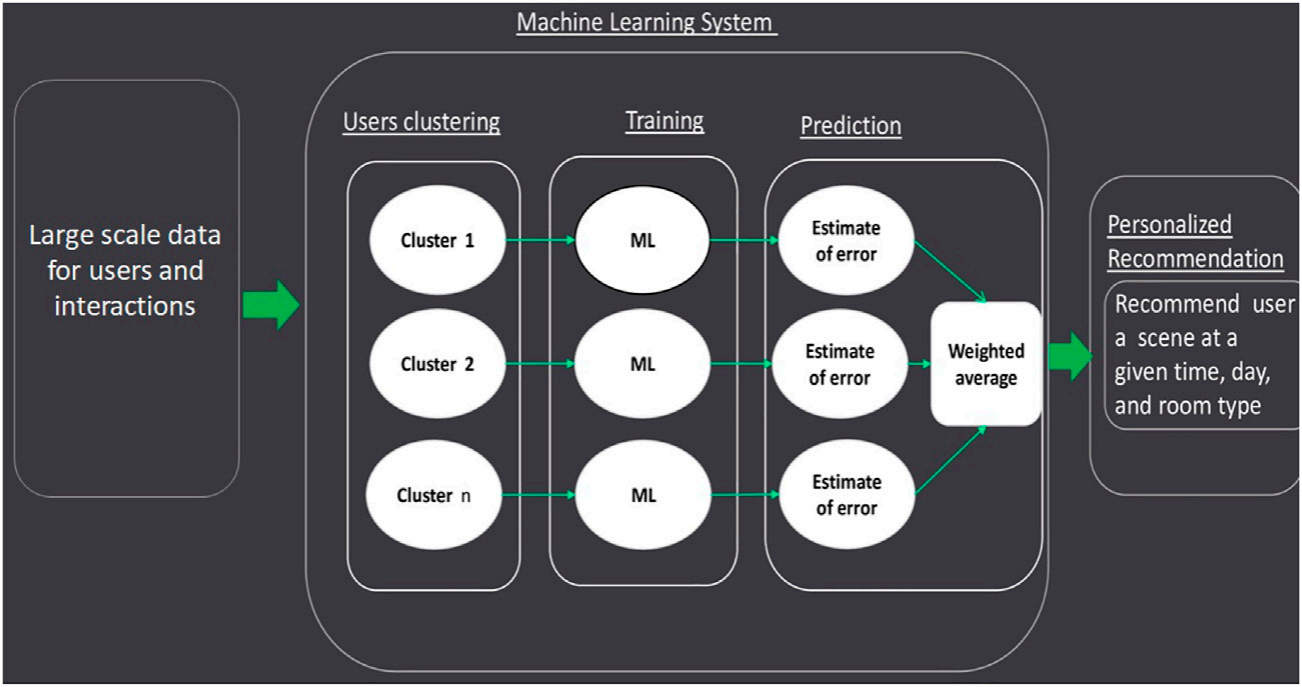
Proposed methodology for scene prediction.

The scene recommendation methodology is shown in Figure 2. In the scene recommender system, we cluster users based on similar characteristics and identify the most probable used scene in each hour based on the user clusters. Our sample consists of users located in four different countries across the world. We consider two highly used room types. We first cluster the users into groups based on their usage patterns and geographical location. Then, using those groups, we train our machine learning model to predict the scene usage within each group of similar users. Finally, we report the overall prediction accuracy via a weighted average over all clusters. We use multiple machine learning algorithms to explore the method before and after the clustering described above, and we present detailed descriptions of the methods in each section below.

### 3.4 Data Description and User Clustering

**FIGURE 3:**
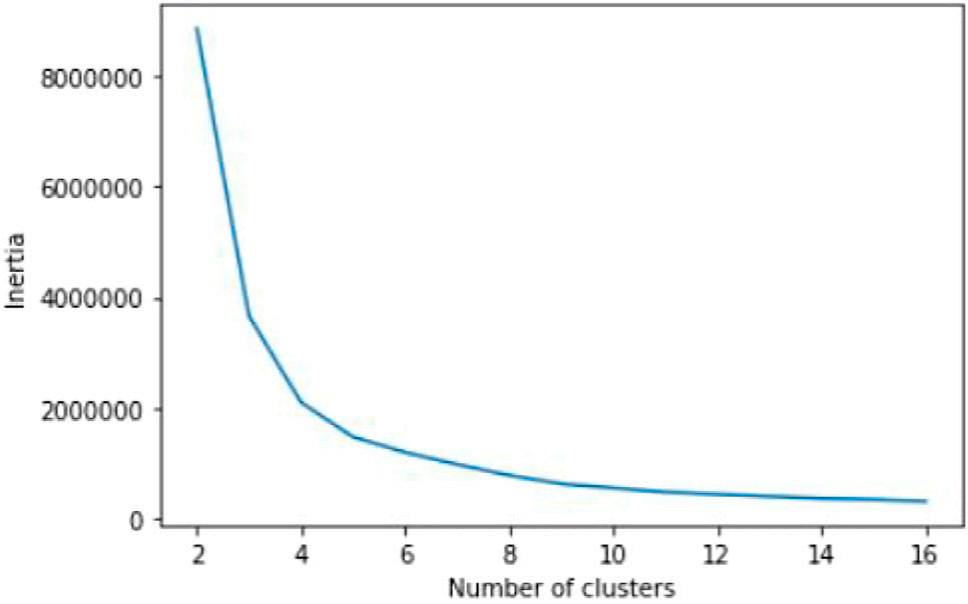
Optimum value of number of cluster.

Our sample includes a total of 578 households located in United States and abroad *b* ∈ *B*. This analysis considers two highly used room types as *r* = {*r*∣*r* ∈ (*room*1, *room*2)} . The analysis is at the room level and therefore, all the scheduling, color recommendations and predictions are done at the room level for each room type. As a result, each household can have a maximum of two room types in our analysis. Our period of study was the 2019 calendar year, and the analysis is based on eight predefined color environments referred to as “scene” in this analysis *s* ∈ *S* . Clustering of similar users is based on usage characteristics and geographical locations for each room type. We consider (0.15–0.85) quantiles values of “avg turn on frequency” defined in Eq. 2, a vector of [1440 ∗ 1] dimension (for 24 h* 60 min) and one-hot encoding of country and room types as features for clustering. The number of clusters is defined using the elbow method over inertia values, which resulted in three clusters, corresponding to elbow point in Figure 3.

**FIGURE 4:**
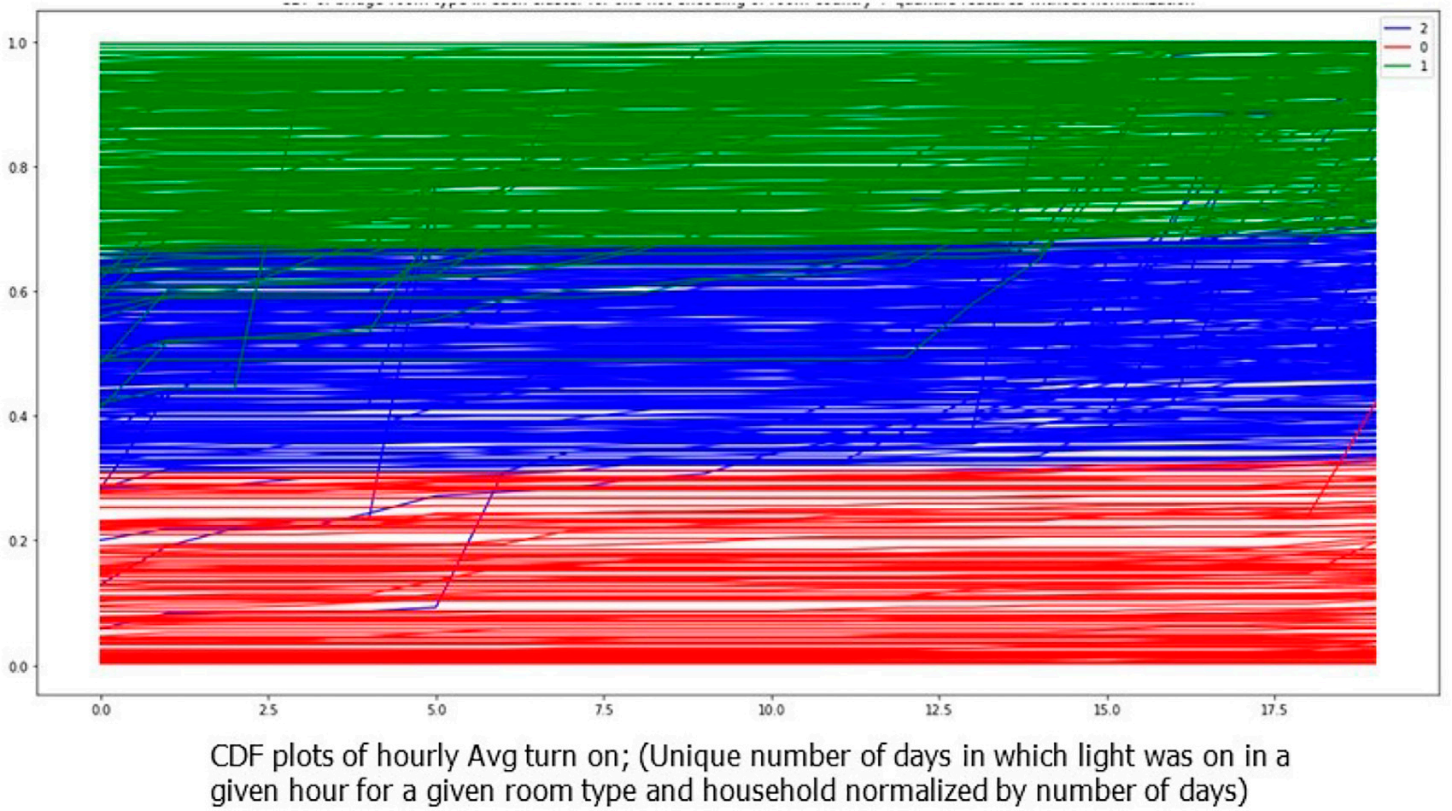
CDF plots for each household-room type colored by cluster number.

Later, to show the effectiveness of the clustering method, The distribution of system usages in each household and room type is plotted. The CDF plots in Figure 4 show the effectiveness of the method, as CDF lines are close to each other within each cluster and are separated from the other clusters.

### 3.5 Features

**FIGURE 5:**
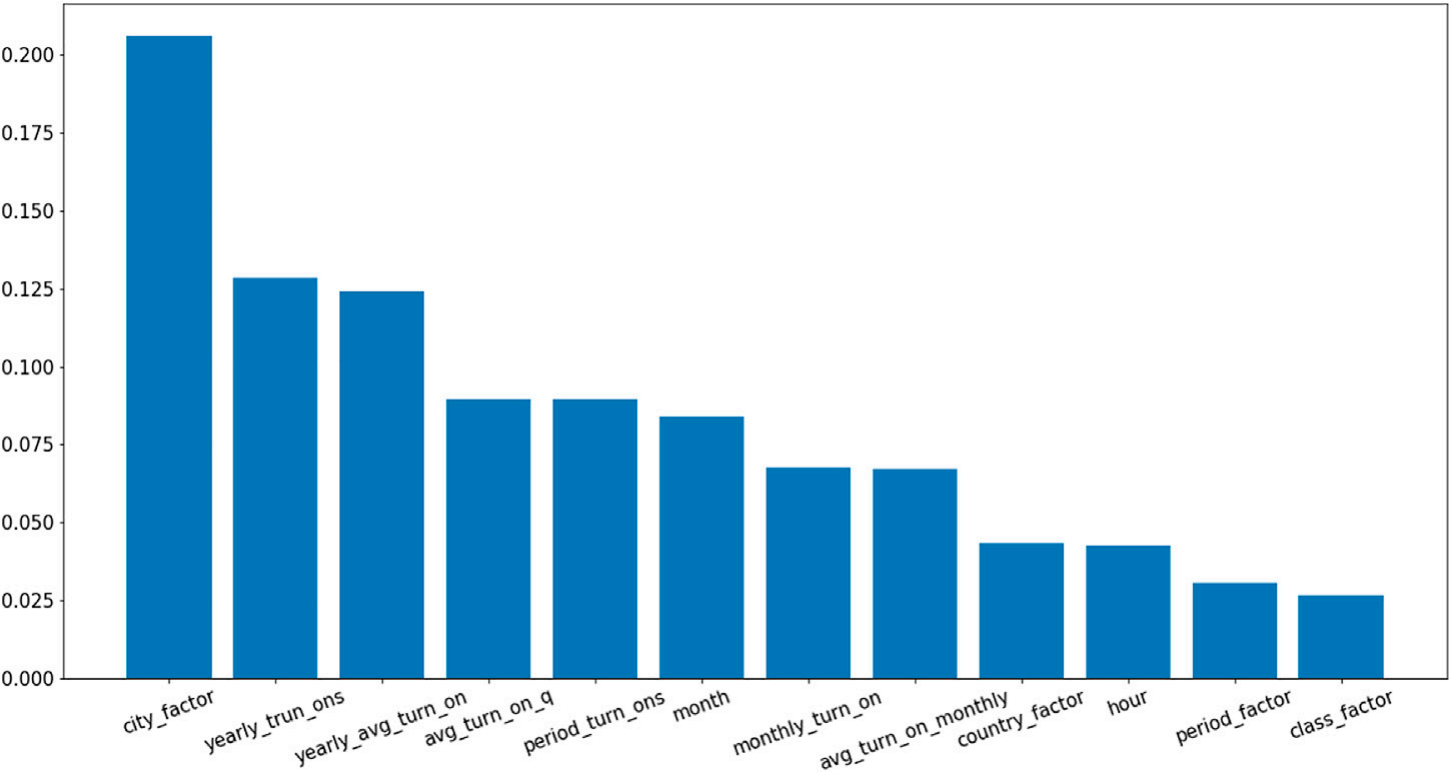
Feature importance.

Considering the methodology presented in Figure 2, a numerical description of the features are presented in Table 1. The importance of each feature is presented in Figure 5. The description of features used in both Figure 5; Table 1 are as follows:

1 Monthly turn on: the unique number of days that light was on in a given hour for a given room type and a particular household.
2 Avg turn on: normalizes monthly turn on by dividing it to the number of days available in 1 month.
3 Quarterly turn on: the unique number of days light was on in a given quarter in a given hour for a given room type and particular household.
4 Avg turn on quarterly: normalized quarterly turn on divided by the number of days available in a quarter.
5 Yearly turn on: the unique number of days light was on at a given hour for a given room type and particular household.
6 Yearly avg turn on: normalized yearly turn on divided by the total number of days in the year.

**TABLE 1:** Numerical description of features.

	month	hour	Monthly turn on	Avg turn on monthly	Quarterly turn on	Avg turn on quarterly	Yearly turn on	Yearly avg turn on
Mean	5.83	11.5	22.1	0.74	61.52	0.68	219.26	0.68
Std	3.09	6.92	8.42	0.28	23.64	0.26	71.19	0.22
Min	1	0	1	0.03	1	0.01	1	0.00
25%	3	6	16	0.53	44	0.49	170	0.52
50%	6	12	25	0.83	65	0.72	228	0.70
75%	8	18	30	1	84	0.93	283	0.87
Max	11	23	31	1.03	92	1.02	324	1

We also consider temporal characteristics such as “month,” “hour” and “period factor.” Geographical features are presented as “city factor” and “country factor.” Finally “class factor” defines the room type in this analysis.

### 3.6 Training and Prediction

We utilize random forest, KNN, and XGBoost to compare the prediction performance across different classes. Random forest is a tree-based meta estimator that fits an estimator on various sub samples, reduces the prediction variance and prevents overfitting by averaging over the models trained on the sub samples. Sub sample size is controlled with the bootstrap option. XGBoost is an ensemble method of gradient boosted trees, and it works by combining weak predictive tree models and learning from them. KNN implements k nearest neighbors and voting among neighbors. In this study, the above mentioned methods’ parameters were optimized based on grid search on the following hyperparameters: number of trees, max depth of trees, and number of neighbors. We split the data to train-test split and use independent test and cross validation for evaluating the predictions.

### 3.7 Evaluation

Here we analyze the performance of different algorithms described in the methodology, before and after the clustering. Using data described in section 3.4 and features described in section 3.5 we aim to provide a personalized scene recommendation. To do so, we first compare different algorithms and their prediction performance for each of our classes (colors). We used precision, recall, F-score, accuracy and balanced accuracy in our framework for evaluation purposes in Equations (recall-balanced ac).

• (TP):True Positive
• (TN):True Negative
• (FP):False Positive
• (FN):False Negative

$$Recall=\frac{TP}{TP+FN}$$ (3)

$$Specificity=\frac{TN}{TN+FP}$$ (4)

$$Precision=\frac{TP}{TP+FP}$$ (5)

$$Accuracy=\frac{TP+TN}{TP+FP+TP+TN}$$ (6)

$$F1-score=\frac{2\;*\left(Precision\;*\;Recall\right)}{Precision+Recall}$$ (7)

$$Balanced\;Accuracy=\frac{Recall+Specificity}{2}$$ (8)

## 4 Results and Discussion

### 4.1 General Recommendation

In this study, we analyzed the performance of each category (color) in our multi category classification problem. We then provided a summary result for each method, both in terms of accuracy and balanced accuracy. Later, by selecting the algorithm, we compared the performance of the algorithm for the two phases of before and after clustering. Moreover, we analyzed the accuracy and balanced accuracy within each separate cluster. The results of our model are described in this section in three stages: First, prior to clustering, we set a benchmark by analyzing different algorithms and their performance on our set up. Second, we summarized the results of classifier estimates into two single metrics of accuracy and balanced accuracy. Third, we trained our algorithm on each cluster separately, reported the accuracy and balanced accuracy results, and aggregated them.

In the first stage, we randomly split the data into train and test (90–10%) sets. Table 2 shows the results of different algorithms in terms of recall, precision, and F-score metrics on our test set using each of the classification methods discussed in section 3.6.

**TABLE 2:** Performance metrics in different algorithms (test set).

	Precision			Recall			F-score		
Class/method	RF	KNN	XGBoost	RF	KNN	XGBoost	RF	KNN	XGBoost
0	0.97	0.94	0.92	0.97	0.96	0.95	0.97	0.95	0.93
1	0.97	0.94	0.93	0.91	0.94	0.93	0.94	0.94	0.93
2	0.94	0.90	0.94	0.88	0.87	0.88	0.91	0.89	0.91
3	0.96	0.93	0.96	0.99	0.91	0.91	0.97	0.92	0.93
4	0.96	0.87	0.95	0.94	0.81	0.94	0.95	0.84	0.95
5	1.00	0.92	0.96	1.00	0.81	1.00	1.00	0.86	0.98
6	0.97	1.00	0.97	0.89	1.00	0.91	0.93	1.00	0.94
7	1.00	0.84	1.00	1.00	0.79	1.00	1.00	0.82	1.00
8	1.00	0.71	1.00	0.87	0.71	1.00	0.93	0.71	1.00

The scene prediction problem is a multi category classification problem and, therefore, classifier performance should be judged based on each separate category. The category prediction results presented in Table 2 confirmed that there was no crucial imbalance in the prediction. Moreover, performance was relatively robust with respect to each category. In the second stage, classifier estimate results provided for each category in Table 2 were summarized into two single metrics (accuracy and balanced accuracy) as shown in Table 3.

**TABLE 3:** Test accuracy before clustering (test set).

Method	Accuracy	Balanced accuracy
RF	0.965	0.939
KNN	0.9312	0.86
XGboost	0.928	0.946

In the third stage, using features and methods described in section 3.4, we group our sample into three clusters. Considering F1-score and accuracy, we used random forest as our classifier. Within each cluster, we split our data into train-test (90–10%) sets and reported the results in terms of accuracy, balanced accuracy and weighted average accuracy over the clusters populations in Table 4. As shown in Table 4, the overall weighted average has a meaningful increase both in terms of accuracy and balanced accuracy. This indicates that training within similar users enhances the recommendation system by 0.72 percent in terms of accuracy.

**TABLE 4:** Weighted test accuracy after clustering.

Cluster number	Balanced accuracy	Accuracy	Cluster population
0	0.98	0.987	133
1	0.93	0.972	259
2	0.94	0.965	263
Overall weighted average	0.944	0.972	655

### 4.2 Cold Start

**FIGURE 6:**
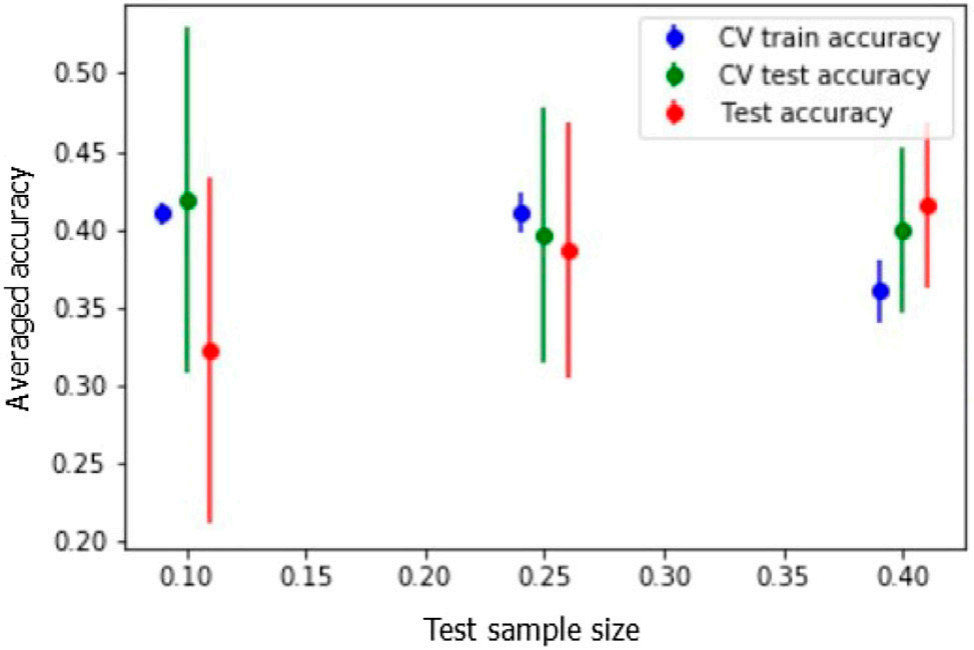
Random sampling on household separation result.

The lack of prior knowledge on individual user preferences causes recommendation systems to face a problem known as cold start. Here we analyze cold start in our scene prediction problem and we report our efforts to cope with this issue. We show that clustering can increase the prediction accuracy in a cold start context. Figure 6 shows the effect of sampling size on model performance when facing cold start. The train and test sets include different households and therefore, it reflects the condition of no prior knowledge on specific household preferences.

We investigated three test sets with different sample sizes as follows: In scenario 1, we reserved 10% of households for the test set and trained on 90%. In scenario 2, we tested on 25% of households and trained on 75%. In scenario 3, we reserved 40% of households for the test set and trained on 60%. We ran 20 randomly sampled iterations using this set up for each scenario and reported cross validation averaged accuracy on train and test sets. In addition, we obtained independent test prediction accuracy as shown in Figure 6. The average accuracy over the independent test prediction and test cross validation increase by increasing the sample size. Moreover, the increase in training sample size results in lower deviation in test accuracies for both cross validation and independent test prediction. Cross validation shows better performance in terms of average test set accuracy in the first scenario where the test size is smaller. As with smaller datasets it is better to do cross validation and train on the entire dataset. On the other hand, when running independent testing, the average accuracy increases by increasing the training sample size. As such, by increasing the test sample size to 40% we achieved a more robust model. Overall, the comparison of cross validation accuracy in train sets in the three scenarios suggests that having more data would result in better performance in terms of accuracy and with smaller dataset deviation as prediction accuracy increases.

Table 5 shows the effect of clustering on how well we can predict the preferences in scene recommendations for the cold start problem. As shown, after clustering, accuracy has a meaningful increase and is beyond the classifier’s deviation. It is notable that accuracy in clustering method is reported by a weighted average over the population of each cluster.

**TABLE 5:** Cold start problem and clustering.

Clustering	Metric	Mean	Standard deviation
Before	CV test	0.41	0.03
After	CV weighted average	0.44	0.02

It should be noted that many personal and environmental factors affect lighting needs and preferences in each room type and household. The personal factors include mood, general activity, personality, and living style while the environmental factors include design of building for each room type, apartment level, window size and direction, and level of daylight for each room. When there is no prior knowledge of users’ environmental and personality factors, a time-location based prediction of users’ desired light color can be challenging. Thus a more accurate prediction would require access to personal information that could interfere with user privacy.

## 5 Conclusion and Future Work

Energy consumption is a crucial factor in house lighting products, and smart lighting has a high potential for energy saving Von Neida et al. (2001). However, a recent study showed that energy consumption was not an influential factor among smart lighting users Zarindast et al. (2021a) and they consider ease of use as an important contributing factor Zarindast et al. (2021a). Moreover, ease of use and perceived usefulness are significant factors in smart lighting purchase intention Shin et al. (2018). As a result, since a personalized recommendation feature in smart lighting products can contribute to the ease of use aspect of lighting systems, it can contribute to the adoption rate of smart lighting products.

To increase the functionality of the smart lighting system for households, ease the use of the system and increase the control ability of the system, this paper introduced a light routine and color recommendation system for household residents. This problem consisted of two subproblems: light schedule and color, which we dealt with as routine and scene recommendations. We proposed separate frameworks for each subproblem as follows: For routine recommendation, we used an unsupervised clustering method based on the frequency of light usage in each minute of the period of analysis. The most frequently used time period would be suggested as a light routine to the user. We did feature engineering and trained and predicted light color using random forest, KNN and XGboost. Based on users’ usage characteristics and geographical location, we clustered them and used these clusters to train our machine learning algorithms. We considered the scene recommendation problem as a multi-category classification problem. We leveraged historical data from households located in different geographical locations worldwide for 1 year to develop personalized recommendations. We utilized clustering to enhance our system’s prediction performance and we analyzed the performance of our classifier on two different settings (with and without prior knowledge of household preferences). Results revealed that training on similar users enhanced our classifier’s prediction performance on generic and cold start recommendations.

Mood and emotion affect how humans perceive and interact with lighting systems. Therefore, identifying human mood, behavior, psychology, and emotions at each time period using other sources and associating those pieces of information with a recommendation system is a promising line of future research work. Another potentially useful line of research would be investigating and quantifying the effect of smart lighting on human psychology, building management systems, etc.

## Data Availability

The datasets presented in this article are not readily available because of company restrictions. Requests to access the datasets should be directed to atousa, atousaz@iastate.edu.
